# Cancer and cardiovascular disease: can understanding the mechanisms of cardiovascular injury guide us to optimise care in cancer survivors?

**DOI:** 10.3332/ecancer.2022.1430

**Published:** 2022-07-14

**Authors:** Lan-Linh Truong, Laura Scott, Raveen S Pal, Mathew Jalink, Sanjeeva Gunasekara, Don Thiwanka Wijeratne

**Affiliations:** 1 Department of Medicine, Queen’s University, Kingston, ON K7L 3N6, Canada; 2 Department of Public Health, Queen’s University, Kingston, ON K7L 3N6, Canada; 3 National Cancer Institute, Maharagama 10280, Sri Lanka; 4 Sri Lanka Cancer Research Group, Colombo 10230, Sri Lanka; 5 Division of Cancer Care and Epidemiology, Queen’s University Cancer Research Institute, Kingston ON K7L 3N6, Canada

**Keywords:** chemotherapy-induced cardiotoxicity, cardiovascular disease, cancer survivors, cancer survivorship, chemotherapy-related cardiac dysfunction, heart failure, coronary artery disease, primary prevention, secondary prevention, cardio-oncology

## Abstract

Cancer and cardiovascular disease (CVD) are the leading causes of morbidity and mortality. Therefore, CVD deaths in cancer survivors remain a major challenge in improving cancer outcomes, especially in low and middle income countries (LMICs). Cancer and CVD share many common risk factors, both modifiable risk factors (obesity, diabetes and smoking) and non-modifiable factors such as inflammation. Additionally, some cancer therapies are associated with cardiac toxicity. These mechanisms drive increased CVD outcomes in cancer survivors, and understanding this relationship allows us to target therapies to combat such risks. Several commonly used pharmacotherapies for CVD demonstrate promise in cancer survivors for both primary and secondary prevention. Beta blockers and Angiotensin converting enzyme (ACE)-inhibitors have been shown in several studies to improve left ventricular ejection fraction (LVEF) in patients with already established LVEF decline following cancer therapy. Statin use during chemotherapy was associated with lower risk of heart failure and smaller declines in LVEF. Recent studies into the effects of anti-inflammatory medications on cardiovascular events in the non-cancer population have demonstrated promising results and may prove to be an area of further investigation and possible benefit in the cancer population [Canakinumab Anti-Inflammatory Thrombosis Outcomes Study (CANTOS) and Colchicine Cardiovascular Outcomes Trial (COLCOT)]. Additionally, several other medications including PCSK9 inhibitors, sodium-glucose cotransporter-2 inhibitors (SGLT2i) and glucagon-like peptide 1 (GLP-1) agonists have been shown to modify inflammation, and therefore may provide cardiovascular benefits. While common pharmacotherapies used in CVD show promise in cancer survivors, their exact mechanisms remain poorly understood. Few studies evaluate their clinical effectiveness specifically in cancer survivors, as this patient population is excluded from most studies. Further investigation is warranted with more representation of cancer survivors before cost-effective recommendations are made. This is especially true in LMICs where resources are sparse for primary and secondary prevention in order to optimise care in this unique, high-risk population for CVD.

## Introduction

Cardiovascular disease (CVD) and cancer account for nearly two-thirds of all non-communicable disease deaths globally [1]. CVD and cancer have long been shown to be leading causes of morbidity and mortality in high income countries and are increasingly becoming leading causes of morbidity and mortality in Low and Middle Income Countries (LMIC) due to alterations in demographics and socio-economic characteristics, as well as a lack of access to medical care in this population [2]. Despite high mortality rates, recent advancements in cancer therapeutics have led to significant improvements in cancer survival, with an estimated 14 million cancer survivors in the United States in 2014 and a projected 19 million by 2024 [3]. CVD is the leading cause of non-cancer mortality among cancer survivors, with the majority of CVD mortality being attributed to either ischaemic heart disease or congestive heart failure (CHF) [4, 5]. In recent years, there have been incredible advancements in cancer treatment; however, there remains an opportunity to further improve survival outcomes in cancer survivors through the prevention and treatment of CVD and its risk factors. The objective of this narrative review is to explore the pathophysiology behind and clinical consequences of CVD in cancer survivors, and the prevention and treatment of CVD in this population. We investigated the shared risk factors that exist between cancer and CVD, the role of surveillance of CVD in cancer survivors and the evidence of pharmacotherapies in management of cancer and cancer-therapy related CVD. We searched the Embase and Medline databases for relevant articles published in the last 10 years (January 2012 to December 2021) using the search terms related to Cardiovascular, Cancer and Pharmacotherapy.

## Shared risk factors in cancer and CVD

The development of CVD amongst cancer survivors is likely caused by shared risk factors between the two conditions including: lifestyle factors, cancer-associated inflammation and the iatrogenic effects of cancer therapy. Cancer and CVD share many modifiable lifestyle risk factors including: tobacco use, obesity, sedentary lifestyle, poor diet and excessive alcohol intake [6]. Comorbidities are more common in cancer survivors, who when compared to healthy controls are more likely to have hypertension (59.5% versus 65.9%, *p* < 0.01), diabetes (21.5% versus 23.4%, *p* < 0.01), be overweight/obese (35.4% versus 43.4%, *p* < 0.01) and have a history of smoking (21.2% versus 32.7%, respectively, *p* < 0.01) [7]. The presence of these risk factors may translate to atherosclerosis and CVD [7].

Inflammation, both preceding and as a result of cancer, is a risk factor for CVD. Over 25% of cancers are linked with increased inflammatory states, whether it be infection, chronic inflammation or autoimmunity [8, 9]. For example, inflammatory bowel disease is known to increase risk of colorectal cancer (CRC) by 10-fold, and management of colitis with anti-inflammatory therapy reduces this risk [10, 11]. Mechanisms of inflammation seen in patients with both cancer and CVD include high levels of chronic inflammation, oxidative stress, altered telomere length and clonal haematopoiesis of indeterminate potential (CHIP) [12, 13]. From a cardiovascular perspective, CHIP is associated with accelerated atherosclerosis, as genes encoded by CHIP are also involved in de-activation of interleukin-6 (IL-6), ultimately leading to increased circulating levels of IL-6 and increased inflammation [14, 15]. Systemic inflammation caused by the malignancy itself and inflammation associated with cancer therapeutics have also been associated with increased CVD and accelerated development of coronary artery disease (CAD) [16, 17].

Cancer therapies can also be associated with the development of cardiac disease [18]. Traditional cytotoxic chemotherapy, such as anthracyclines, have been associated with a variety of cardiovascular side effects. Anthracyclines, for example doxorubicin, are associated with significant, irreversible left ventricular (LV) dysfunction due to decreased LV wall thickness, mass and LV fractional shortening [19–22]. While higher cumulative doses of anthracyclines (>300 mg/m^2^) have been associated with increased risk of cardiotoxicity [23], subclinical echocardiographic abnormalities have been seen in patients treated with lower cumulative doses (<100 mg/m^2^) [24]. Up to half of survivors treated with anthracyclines experience some form of cardiac dysfunction within 20 years of treatment, causing them to be vulnerable to cardiac dysfunction at younger ages compared to the general population [25, 26]. In more recent years, there have been substantial developments in cancer related therapies, in particular the development of immunotherapy. While immunotherapy has proved to be effective in the treatment of cancer, some agents have also been noted to have cardiovascular side effects. Immune checkpoint inhibitors have been associated with the development of myocarditis, which has been found to be fatal in up to 50% of cases [27, 28]. Chemotherapy related cardiac dysfunction (CTRCD), defined as a >10% drop in left ventricular ejection fraction (LVEF) from baseline, or an absolute value of 53% or less, is a known complication of various cancer therapies (Trastuzumab, Pertuzumab) [29]. CTRCD occurs due to the medication’s interaction with DNA, inhibiting cell replication and subsequent myocyte death [19]. Other cardiotoxic effects include dilated cardiomyopathy, restrictive cardiomyopathy, myocardial infarction, conduction defects, valvular disease, pericardial disease and hypertension [23, 25, 30].

Mediastinal radiation has long been associated with an increased risk of cardiac morbidity and mortality [31]. Cancer survivors who underwent mediastinal irradiation during childhood have significant coronary vessel damage [32]. Almost half had mean coronary artery calcium (CAC) scores of patients 15 years older than their age, although they were often asymptomatic secondary to radiation-related nerve impairment or reduced exercise tolerance [32, 33]. The extent of CAD after mediastinal irradiation is dependent on multiple factors including radiation dose, patient age at the time of radiation, radiation field and time interval since radiation, in addition to the patient’s own personal risk factors [32]. Patients undergoing radiation therapy had a 2.3-fold higher risk for atherosclerotic disease [Odds ratio (OR): 2.3; 95% confidence interval (CI): 1.04–5.3]; and patients undergoing both radiation and chemotherapy had a 4.8-fold increased risk (95% CI: 1.6–14.4) compared to those undergoing surgery alone [34]. Other implications of radiation therapy on the heart include pericarditis, cardiomyopathies and valvular heart disease [35].

The shared risk factors between CVD and cancer are an important area for further investigation, as they may provide opportunities for targeted therapies to reduce the burden of CVD in the cancer population. Additionally, awareness of, and further investigation into the mechanisms of the cardiovascular effects of cancer therapies may serve to provide an opportunity for surveillance and earlier recognition of CVD in cancer patients.

## Importance of disease surveillance for CVD in cancer survivors

Screening for cardiotoxicity in cancer survivors is primarily risk-based and is dependent on patient symptoms, cumulative doses of chemotherapy/radiation therapy and the patient’s overall health. Examples of cardiotoxicity screening among cancer patients include both cardiac troponin (cTnT) and N-terminal probrain natriuretic peptide (NT-proBNP), which have been validated as surrogate markers for late LV structural status in long-term survivors of childhood cancer [36, 37]. A study by Lipshultz *et al* [36] found that elevations in serum cTnT during the first 90 days of anthracycline treatment were associated with reduced LV thickness and increased pathologic LV remodelling 4 years later. Similar findings were true for elevated serum NT-proBNP [36]. An additional study by Cardinale *et al* [38] found that elevated TnI, another component of the troponin complex, is a risk marker for future development of reduced LVEF. A normal TnI identified patients at lower risk of developing reduced LVEF. This study also found that patients with a normal TnI had no cardiac damage in the first year following high-dose chemotherapy, thus helping separate patients in which close monitoring of cardiac function is required [38].

Studies looking at myocardial strain on imaging have shown a high prevalence of abnormal longitudinal strain in childhood survivors exposed to anthracyclines and/or chest radiotherapy, despite having preserved systolic function [39]. The SUCCOUR (Strain Surveillance of Chemotherapy for Improving Cardiovascular Outcomes) study compared the use of global longitudinal strain (GLS) compared to LVEF as a measure of LV dysfunction to initiate cardioprotective therapy in patients at risk of CTRCD. Researchers noted that at 1-year follow-up, the LVEF was less in the Ejection Fraction (EF)-guided arm compared to the GLS-guided arm (55% ± 7% compared to 57% ± 6%, respectively, *p* = 0.050) [40]. Routine surveillance with echocardiography is recommended in cancer survivors with high risk for cardiomyopathy, beginning within 2 years after exposure and repeated a minimum of every 5 years thereafter [41]. This recommendation was made after an international collaboration to harmonise existing cardiomyopathy surveillance recommendations in North America and across Europe. The Children’s Oncology Group, a National Cancer Institute supported clinical trials group, recommends surveillance echocardiograms every 1, 2 or 5 years for survivors of childhood cancer, depending on the presence and degree of three risk factors: age at treatment, cumulative anthracycline dose and whether patients received chest radiation [42]. Chest radiation is defined as any radiation in which the heart was in the field of treatment, including mediastinal, thoracic, spinal, left or whole upper abdominal or total body irradiation. Although there are no guidelines directed towards risk stratification of adult cancer survivors in North America, the American College of Cardiology (ACC), the American Society of Echocardiography and the European Association of Cardiovascular Imaging recommend screening echocardiogram 10 years after treatment, and then at 5-year intervals thereafter [43, 44]. For patients deemed high-risk for radiation-induced heart disease, they recommend screening at 5 years post-treatment and non-invasive stress testing every 5 years [44].

## Supportive pharmacotherapy to reduce CVD in cancer survivors

Given our understanding of the above mechanisms, primary and secondary prevention in cancer survivors is crucial in reducing the burden of CVD. In this section, we explore the role of individual therapies.

### Beta-blockers and renin angiotensin aldosterone system (RAAS) blockade

Beta-blockers (BB) work by blocking the action of endogenous catecholamines on beta-adrenergic receptors, thereby reducing blood pressure, cardiac remodelling and increase filling time with reduced oxygen demand on the heart [45]. The RAAS system regulates blood volume and systemic vascular resistance and its activation triggers sodium reabsorption, shifting fluid into the intravascular space and increasing arterial pressure. The RAAS system also stimulates the release of aldosterone causing further sodium resorption. Additionally, it stimulates thirst, the release of antidiuretic hormone and decreases the sensitivity of the baroreceptor reflex, leading to net increased sodium, total body water and vascular tone [46]. A study by Seicean *et al* [47] demonstrated that breast cancer patients with structurally normal hearts at baseline who were taking BB during treatment with anthracycline (mean total dose 268.3 ± 163.7 mg) or trastuzumab, with or without radiotherapy, had lower incidence of new heart failure (HF) events. The cumulative incidence of HF at 3 years in patients on continuous BB therapy was 2% (95% CI: 0.8–3.2) compared to 9% (95% CI: 8.3–10.3) in control patients [47]. Another study found that patients taking prophylactic BB therapy with carvedilol or nebivolol prior to anthracycline-based chemotherapy had less LV function decline at 6 months (63.8% ± 3.9% from a baseline of 65.5% ± 4.8%) compared to those who received placebo (LVEF 57.5% ± 5.6% from a baseline of 66.6% ± 5.5%) [48, 49]. The Carvedilol for Prevention of Chemotherapy-Related Cardiotoxicity (CECCY) trial found that carvedilol had no impact on the incidence of early onset or LVEF reduction with a 14.5% incidence of cardiotoxicity compared to 13.5% in the placebo group [50].

A randomised controlled trial by Cardinale *et al* [51] investigated cancer patients with elevated cardiac enzymes during treatment, a marker for cardiac injury. Participants were randomly assigned to either receive enalapril or no treatment. At baseline evaluation, LVEF was normal in all patients and comparable in the two groups. After 12 months, 25 control subjects (43%) showed a decrease in LVEF by >10% from baseline compared to no patients in the enalapril group (*p* < 0.001) [51]. In addition, the cumulative number of adverse cardiac events was lower in patients treated with enalapril compared to controls [51]. A separate study by Tallaj *et al* [52] investigated the use of BB combined with RAAS blockade. In patients with chemotherapy-induced cardiomyopathy, those treated with a combination of ACE-inhibitor (ACEi) and BB had significant improvement in LVEF (26% ± 10.20% versus 37% ± 17.6%, *p* = 0.028), which was not seen with Angiotensin converting enzyme inhibitor (ACEi) treatment alone [52]. The SAFE-HEaRt (Cardiac Safety of HER2 Targeted Therapy in Patients with HER2 Positive Breast Cancer and Reduced Left Ventricular Function) study was the first prospective study to demonstrate safety of Human Epidermal growth factor Receptor 2 (HER-2) targeted therapies in patients with reduced cardiac function (LVEF 40%–49%) [53]. 90% of patients (27 of 30) receiving cardioprotective therapy with BB and ACEi/ Angiotensin Receptor Blocker (ARBs) throughout the duration of their HER-2 treatment completed planned oncologic therapy without developing a cardiac event or asymptomatic decline in LVEF, thus proving that collaboration between cardiology and oncology can allow for this specific patient population to achieve optimal cancer therapy while minimising the risk of poor cardiac outcomes [53].

The OVERCOME (Prevention of Left Ventricular Dysfunction with Enalapril and Carbedilol in Patients Submitted to Intensive Chemotherapy for Treatment of Malignant Hemopathies) study demonstrated that concomitant treatment with enalapril and carvedilol can prevent LV systolic dysfunction, with a mild 3.1% absolute difference in the mean LVEF between the intervention and control groups [54]. A similar effect was seen in breast cancer patients with suspected trastuzumab-induced LV dysfunction. Once HF symptoms and LVEF were stable following treatment, 25 women who were given ACEi and BB were re-challenged with trastuzumab. Of them, 22 (88%) had stable LVEF without HF symptoms during follow-up, thus speculating that the combined use of ACEi and BB can lead to LV systolic recovery [55]. The MANTICORE (Multidisciplinary Approach to Novel Therapies in Cardio-Oncology Research) study however, found that while prophylactic ACEi and BB were associated with smaller declines in LVEF, it did not prevent concurrent LV remodelling, the primary outcome measure of this study [56].

In addition to LV systolic recovery, BB have been shown to reduce cancer-related death, reoccurrence and metastasis [57]. A retrospective study including a sample size of 466 consecutive patients with operable breast cancer and a follow-up period of >10 years demonstrated that patients taking a BB had a significantly reduced risk of developing metastasis and tumour recurrence and had longer disease-free survival [58]. Breast cancer patients treated with propranolol are significantly less likely to present with advanced disease and have lower cumulative probability of breast cancer-specific mortality compared with matched non-users [59]. A similar effect was seen in a study which included 24,238 patients with head and neck, lung, ovarian, gastric, colon and prostate cancers, where patients using propranolol had a 25% reduction in cancer recurrence compared to non-propranolol users [60]. This is attributed to the ability of Beta-adrenergic signalling to regulate immune responses to tumour cells, the inhibition of apoptosis and the induction of vascular endothelial growth factor [61–63].

Sacubitril/Valsartan is recommended in current guidelines for patients with heart failure with reduced ejection fraction (HFrEF) to reduce mortality and hospitalisations, but there remains limited evidence in its effectiveness in patients’ concurrent cancer and HFrEF. One study found significantly improved LVEF, with 8 of the included 67 patients’ LVEF normalising. They also noted a significant reduction in NT-proBNP levels and improvement in exercise tolerance, as indicated by change in New York Heart Association (NYHA) functional class [64]. A separate study that included 635 patients noted similar findings, with baseline median NT-proBNP 997.5 pg/ml (InterQuartile Range (IQR): 663.8–2,380.8), which decreased to a median of 416.5 pg/ml (IQR: 192.0–798.2) with *p* < 0.001 [65]. They also found improvements in baseline NYHA functional class and increased LVEF from 26.7% ± 5.4% to 32.3% ± 5.5% (*p* < 0.001) [65].

Sacubitril/Valsartan is recommended in current guidelines for patients with heart failure with reduced ejection fraction (HFrEF) to reduce mortality and hospitalisations, but there remains limited evidence in its effectiveness in patients’ concurrent cancer and HFrEF. One study found significantly improved LVEF, with 8 of the included 67 patients’ LVEF normalising. They also noted a significant reduction in NT-proBNP levels and improvement in exercise tolerance, as indicated by change in NYHA functional class [64]. A separate study that included 635 patients noted similar findings, with baseline median NT-proBNP 997.5 pg/ml (IQR: 663.8–2,380.8), which decreased to a median of 416.5 pg/ml (IQR: 192.0–798.2) with *p* < 0.001 [65]. They also found improvements in baseline NYHA functional class and increased LVEF from 26.7% ± 5.4% to 32.3% ± 5.5% (*p* < 0.001) [65].

### Aspirin

Aspirin is an antiplatelet agent that has long been the mainstay for both secondary prevention of Myocardial Infarction (MI) and stroke [66]. It acts by inhibiting cyclooxygenase (COX) activity, thus inhibits the synthesis of prostanoids, which are involved in modulating inflammatory responses, gastrointestinal cytoprotection and ulceration, atheroprotection and haemostasis among many other functions [67, 68]. Aspirin is administered at low doses to preferentially affect platelet COX-1 activity, but also has some COX-2 activity [69]. In terms of cancer progression, multiple studies have shown that aberrant COX-2 expression is a contributing factor in promoting CRC, with increased expression allowing for elevated prostanoid biosynthesis, and in turn, contribution to the initial steps of tumorigenesis [70]. Overexpression of COX-2 also increases cell migration and proliferation in intestinal epithelial cells [71].

Use of aspirin in primary prevention is currently not recommended in guidelines for conventional diabetic and hypertensive populations; however, cancer patients often have augmented risks of atherosclerosis. A meta-analysis found that aspirin was associated with a reduction in adverse cardiovascular events, but was also associated with an increased risk of major bleeding [72]. Its role in CRC specifically has been evaluated, where it was found to be effective in reducing the risk of CRC-related death in Randomized control trial (RCTs), thus suggesting that the antiplatelet action contributes to the prevention of both atherosclerosis and cancer [73, 74]. As such, the decision to start a patient on aspirin would require assessment of each patient’s risk–benefit profile [75]. The American College of Cardiology (ACC) recommends calculating 10-year CVD risk for patients aged 40–79 [66]. The Coronary artery calcium (CAC) score that is calculated from a cardiac Computed tomography (CT) may have added utility in predicting a nearly 10-fold increase in CVD events in patients with elevated calcium scores [76]. Using CAC score, and distribution of calcification amongst the coronaries, could help guide therapy to higher-risk patients especially those patients with premature atherosclerosis in cancer survivors [77, 78].

The medications discussed below have limited evidence on the utility in cancer survivors with very little reference to cancer populations. We discuss some of the potential benefits that may be applicable to these patients that warrant further study.

### Lipid lowering medication

#### Statins

Statins have long been used in management of hypercholesterolaemia by inhibiting the rate-limiting enzyme involved in cholesterol synthesis [79]. The intermediate products involved in cholesterol synthesis activate various downstream signalling pathways, including regulation of inflammatory cytokines and chemokines [80]. Therefore, beyond cholesterol reduction, statins also decrease oxidative stress, inflammation and the number of inflammatory cells in atherosclerotic plaques [80–82]. With these considerations, it is suggestive that statins can be considered another mechanism to protect against cardiotoxicity induced by cancer and cancer therapies.

Several small, prospective studies have evaluated the effect of atorvastatin on inflammatory markers in patients with LV systolic dysfunction with most noting a significant decrease in the concentration of inflammatory markers in patients with ischaemic and non-ischaemic cardiomyopathy [83–85]. A cohort study demonstrated that women treated with concomitant statins during anthracycline-based chemotherapy had lower risk of HF [Hazard ratio (HR): 0.3; CI: 0.1–0.9; *p* = 0.03] compared to those in the non-statin treated comparison group [82]. Another noted that statin therapy resulted in smaller declines in mean LVEF compared to those not using a statin (−1.3 [3.8%] versus −7.9% [8.0%]) [86]. In terms of LV remodelling, one study noted a significant decrease in LV end-diastolic dimension (57.1 mm to 53.4 mm), compared to patients in the placebo group, who experienced an increase in LV end-diastolic dimension (56.1 mm to 60.3 mm) over a 12-month period [83]. The results of these studies are promising and suggest that statins may have a beneficial effect on CVD in individuals with cancer; however, further investigation is needed to determine the extent of the possible benefit of statins in this population.

#### PCSK-9 inhibitors

Proprotein convertase subtilisin/kexin type 9 serine protease (PCSK9) encodes a protein that prevents removal of low density lipoproteins (LDL) particles from the blood stream, thus PCSK9 inhibitors lead to decreased LDL concentrations and reduced risk of CVD [87, 88]. PCSK9 is also expressed in tissues including the brain, kidney and vascular wall [89]. In smooth muscle cells, it directly increases inflammation via the Nuclear Factor kappa B (NF-kB) pathway, thus promotes plaque monocyte infiltration and macrophage inflammation, and in turn contributing to the development of atherosclerosis [90, 91]. Interventions using monoclonal antibodies (MABs) have been the most commonly investigated and have consistently demonstrated significant efficacy in reducing LDL. A study by Shapiro *et al* [92] saw reductions in LDL by approximately 50% when MABs inhibiting PCSK9 were used as monotherapy, and approximately 70% when used in combination with statins and ezetimibe with excellent short-term safety and tolerability profile.

There was a strong correlation between intracellular lipid accumulation and expression of C-C chemokine Receptor type 2 (CCR2), a chemokine receptor involved in monocyte chemotaxis, implying a causal relation between lipid levels and pro-inflammatory changes [93]. As such, the use of PCSK9 MABs was associated with reversing the pro-inflammatory profile of monocytes in patients with familial hypercholesterolaemia, decreased tumour necrosis factor (TNF) release and increased secretion of the anti-inflammatory cytokine IL-10 [93]. This suggests that in addition to its lipid lowering properties, PCSK9 MABs also reduce CVD by reducing the inflammation that underlies atherosclerosis. The component involving inflammation is especially relevant in cancer survivors, given the increased inflammation occurring as a consequence of cancer diagnosis and therapeutics and the relationship between increased inflammation and CVD. Given this same hypothesis, PCSK9 MABs have also demonstrated reduction in cancer risks [94]. At this time, there are no long-term studies evaluating the effect of PCSK9 MABs in reducing CVD risk factors in cancer survivors, though is likely a new emerging area for research.

### Role of antidiabetics

#### Sodium-glucose cotransporter-2 (SGLT2) inhibitors

SGLT2 inhibitors are used in the management of type 2 diabetes mellitus (T2DM) and work by inhibiting glucose reabsorption in kidneys [95]. SGLT2 cotransports sodium (Na) with glucose, such that the inhibition of SGLT2 leads to reduced reabsorption of both glucose and Na, leading to plasma volume contraction, decreased systolic and diastolic blood pressure with cardiovascular and renal benefits [96]. The plasma volume contraction haemodynamically unloads the LV, thereby decreasing myocardial oxygen demand, ventricular wall tension and filling pressures [97]. The Empagliflozin, Cardiovascular Outcomes, and Mortality in Type 2 Diabetes (EMPA-REG OUTCOME) trial found that in patients with diabetes, when compared to those receiving placebo, patients receiving empagliflozin had a 14% reduction in cardiovascular death, nonfatal MI, nonfatal stroke and >30% reduction in cardiovascular mortality, overall mortality and HF hospitalisations. This finding is especially notable, as there was only a marginal difference in HbA1c between the two groups [96]. A meta-analysis by Zhang *et al* [98] including 351,476 patients demonstrated similar results with statistically significant reduction in risk of major adverse cardiac events, all-cause mortality, cardiovascular mortality, nonfatal MI and hospitalisation for HF. SGLT2 inhibitors were also shown to have favourable cardiovascular outcomes in patients with pre-existing HF with both reduced and preserved ejection fraction [99, 100].

Decreased glucose metabolism associated with SGLT2 inhibitors could also help modulate inflammatory processes that contribute to CVD. For example, inflammatory macrophages preferentially use glucose through the glycolysis pathway [101]. SGLT2 inhibitors also improve the differentiation of epicardial adipose tissue, a known source of inflammatory mediators, and thus reduce the secretion of proinflammatory cytokines [102]. As a result, it is hypothesised that the use of SGLT2 inhibitors could reduce inflammatory processes in vascular endothelial cells and also contribute to weight loss thereby reducing the risk of CVD and cancer [101]. These results appear to be encouraging; however, further investigation in the cancer survivor population is warranted to determine the benefit of SGLT2 inhibitors in this population.

#### Glucagon-like peptide 1 (GLP1) agonists

GLP1 is a peptide secreted by enteroendocrine cells primarily involved in stimulating glucose-dependent insulin secretion and incretin signalling, though has also been linked with regulating inflammation and cardiovascular function [103]. Activation of the GLP1 receptor (GLP1-R) leads to inhibition of gastric and small bowel motility, leading to delayed nutrient absorption and reduced appetite. Hence, the use of GLP1 agonists in the management of diabetes has the added benefit of weight loss [104].

In addition, GLP1 is linked with regulating local and systemic inflammation and cardiovascular function [103]. GLP1 levels are shown to be elevated in chronic and acute inflammatory processes such as sepsis or chronic kidney disease, and correlated with the severity of illness and clinical outcomes [105]. The LEADER (Liraglutide and Cardiovascular Outcomes in Type 2 Diabetes) trial, which included 9,340 patients with T2DM, found that the occurrence of major adverse cardiac events was reduced in the liraglutide group [106]. In addition, fewer patients died from cardiovascular causes in the liraglutide group than in the control group [106]. A similar finding occurred in a separate study with 14,752 subjects using either once-a-week administration of exenatide, a long-acting GLP1 agonist, compared to placebo [107].

The added effect of weight loss with GLP1 agonists further reduces cardiovascular risk factors in cancer survivors. Long-term survivors of cancer are more likely to have poor adherence to dietary and physical activity guidelines, and cancer treatment regimens often include corticosteroids, which are known to increase percentage body fat and caloric intake in survivors [108, 109]. Vigorous exercise has been associated with a lower risk of CVD in a dose-dependent manner, independent of clinical and treatment-related risk factors [110]. As previously discussed, cancer therapeutics are known to cause LV dysfunction and in turn, can be attributed to impaired cardiovascular fitness and exercise tolerance [109]. Hence, the combined effect of reducing inflammation and weight loss makes GLP1 agonists a promising medication for the prevention of CVD in cancer survivors who are also diabetic, although they have not specifically been studied in these patients.

### Other medications

#### Dexrazoxane

Dexrazoxane is a chelating agent that binds to intracellular iron before it enters cardiomyocytes, thus decreasing free radical formation and reducing cardiomyocyte apoptosis. It is currently the only Food and Drug Administration (FDA)-approved drug used for the prevention of anthracycline-related cardiotoxicity [111]. Several trials evaluating breast cancer patients undergoing chemotherapy with doxorubicin have shown a decrease in the risk of developing of HF when dexrazoxane was added to treatment [112]. A meta-analysis estimated an overall reduction in cardiac events by 65% while others have estimated a reduction of up to 82% [113]. It has also been shown to prevent cardiotoxicity in children and adolescents treated with anthracyclines [114]. This same study also highlighted that dexrazoxane exhibits its cardioprotective effects without decreasing the effectiveness of anthracyclines or affecting event-free survival [114]. It should be noted however, that the cardioprotective activity of dexrazoxane is not fool-proof as anthracyclines have several mechanisms causing cardiotoxicity, and dexrazoxane offers protection for some, but not all mechanisms of cardiotoxicity [115–117]. This may benefit cancer survivors and is an area that warrants further study.

### Biologics

TNF is a pro-inflammatory cytokine secreted primarily by immune cells and is involved in both inflammation, cell proliferation, apoptosis and lipid metabolism [118]. While anti-TNF agents have been long used in the treatment of autoimmune inflammatory conditions, its role in other inflammatory conditions such as CVD has not been extensively studied. TNF drives inflammation and plaque formation in atherosclerosis and thus its inhibition is a potential target for the prevention of CVD [119]. Multiple observational studies have shown that TNF inhibition reduces atherosclerosis and cardiovascular events when administered to patients with rheumatoid arthritis [120]. Interestingly, TNF levels post-MI are a strong predictor of recurrent events [121]. In addition, higher TNF levels are associated with higher odds of CAD (OR: 2.25, 95% CI: 1.50–3.37) and ischaemic stroke (OR: 0.54, 95% CI: 0.42–0.96) [118].

The CANTOS study, which sought to test the inflammatory hypothesis of atherosclerosis, included patients with a history of MI who were either assigned to receive placebo or a MAB canakinumab, which targets IL-1β, an inflammatory cytokine [122, 123]. Patients taking canakinumab had significantly reduced levels of C-reactive protein (CRP) and IL-6 compared to placebo. Patients receiving a 150 mg dose of canakinumab had 15% lower risk of primary end point (non-fatal MI, non-fatal stroke or cardiovascular death) compared to the placebo group (3.86 versus 4.50 events per 100 person years). Interestingly, cancer mortality was found to be significantly lower in patients receiving canakinumab than those receiving placebo, consistent with the notion that inflammation is a risk factor for cancer morbidity and mortality [8, 123]. It specifically showed a dose-dependent reduction in risk of lung cancer, up to 67% [124]. However, the use of Canakinumab as first-, second- or third-line treatment with chemotherapy in non-small cell lung cancer did not confirm the benefit [125]. Unfortunately, separate trials investigating the use TNFa inhibitors (etanercept and infliximab) in HF were stopped due to worsening CHF and worse prognosis compared to placebo [126, 127]. This mechanism is poorly understood, especially considering that elevated TNFa is consistently associated with CHF. Further investigation is needed to determine that if there is a roll for TNFa inhibitors in the cancer population.

## Conclusion

Our narrative review outlines several strategies in reducing the burden of CVD in cancer survivors. The suggested strategies are based on the notion that CVD and cancer share many common risk factors, both modifiable risk factors and biochemical properties such as inflammation. Multiple cancer therapeutics are also associated with cardiac toxicity. We hypothesise that these three mechanisms are the driving forces behind poor CVD outcomes in cancer survivors, and that this understanding can facilitate therapies to reduce such risks.

Of the various drug classes investigated, several demonstrate promise and may warrant further investigation for potential integration into management of CVD in cancer survivors. The use of BB combined with ACEi was shown in several studies to prevent LVEF decline in patients receiving anti-cancer therapies and improve LVEF in patients who have already suffered LV dysfunction following cancer therapy [47–49, 52, 128]. The use of statins during chemotherapy was associated with lower risk of HF and smaller declines in LVEF [82, 86]. PCSK9 MABs were associated with decreased TNF release and increased secretion of IL-10, an anti-inflammatory cytokine [93]. The use of SGLT2 inhibitors is known to improve cardiovascular outcomes and has been shown to reduce oxidative stress and inflammation [96, 98, 101]. GLP1 agonists, in addition to their ability to manage diabetes, have been associated with weight loss and increased GLP1 levels have been correlated with severity of inflammatory processes [105].

While common pharmacotherapies for CVD show promise in cancer survivors, their exact mechanism remains poorly understood. Only a few studies evaluated the clinical utility of these therapies in cancer survivors, often excluding this very population from most studies. Further investigation with more representation of cancer survivors assessing their clinical outcomes is warranted before recommendations are made for primary and secondary prevention in this unique high-risk population for CVD.

Due to shifting patterns of demographic and socio-economic characteristics in LMICs, CVD and cancer are fast becoming the leading causes of morbidity and mortality [129]. This problem is further exacerbated by limited access to expertise, investigations and medicines needed for treating these patients [2]. Therefore, CVD deaths in cancer survivors are a major challenge in improving cancer outcomes in LMICs. In these countries, adapting risk based cardiac screening protocols for cancer survivors, controlling CVD risk factors in cancer patients and choosing less cardiotoxic treatment regimens might help in compensating for some of these existing care gaps [12, 42, 130].

## Conflicts of interest

The authors in this paper have no conflicts of interest to declare.

## Funding statement

This article was not directly funded.
